# Patients’ perspectives of living with a percutaneous endoscopic gastrostomy (PEG)

**DOI:** 10.1186/1471-230X-12-126

**Published:** 2012-09-18

**Authors:** Lena Martin, John Blomberg, Pernilla Lagergren

**Affiliations:** 1 Unit of Upper Gastrointestinal Research, Department of Molecular Medicine and Surgery, Karolinska Institutet, Norra Stationsgatan 67, 2nd floor, SE-171 76 Stockholm, Sweden; 2 Department of Clinical Nutrition and Dietetics, Karolinska University Hospital, Stockholm, Sweden; 3 Department of Surgical Gastroenterology, Karolinska University Hospital, Stockholm, Sweden

**Keywords:** Experience, Impact, Nutrition, Support

## Abstract

**Background:**

Since enteral nutrition therapy is the preferred nutritional support for dysphagic patients with a range of diagnoses, PEG has become part of traditional care. However, enteral nutrition with PEG transfers treatment responsibility and activity to the patients and their carers, so the advantages should be discussed. The aim of this study was therefore to investigate patients’ experience of living with a percutaneous endoscopic gastrostomy (PEG) in order to increase the understanding of patients’ need for support.

**Method:**

In a prospective study at Karolinska University Hospital in Sweden, data were collected consecutively at the time of PEG and two months later using a study-specific questionnaire about each patient’s experience of living with a PEG. Fishers exact test was used to test for statistically significant difference at five per cent level.

**Results:**

There were 104 responders (response rate of 70%). Women felt more limited in daily activity compared to men (p = 0.004). Older patients experienced a more limited ability to influence the number of feeding times compared to younger (p = 0.026). Highly educated patients found feeding more time-consuming (p = 0.004). Patients with a cancer diagnosis reported that the PEG feeding interfered with their oral feeding more than patients with a neurological disease (p = 0.009). Patients mostly contacted the PEG outpatient clinic with problems regarding their PEG, and were mainly assisted by their spouse rather than district nurses.

**Conclusions:**

PEG feeding is time-consuming and interferes with daily life. Although 73% was satisfied, patients’ experiences of living with a PEG may be dependent on age, sex, education and diagnosis. Spouses are the main carers for PEG patients at home, and patients prefer to go to the PEG outpatient clinic for help if problems occur.

## Background

The growing awareness of the relevance of nutrition support in the treatment of diseases has contributed to a rapid increase in the use of percutaneous endoscopic gastrostomy (PEG) worldwide [1,2]. For patients with preserved intestinal function but with inadequate or no independent oral food intake, enteral nutrition therapy via PEG is one of the preferred alternatives to nutrition support [3]. Appropriate nutritional interventions enable a reduction of surgical complications [4], shorten the recovery time and the length of hospital stay [5], improve tolerance to treatment [6] and even increase the rate of survival [7]. PEG has become a part of traditional care for a range of diagnoses e.g., tumours of the head and neck region or the oesophagus, in the care of the elderly and patients with neurological impairment (e.g. stroke, ALS and multiple sclerosis).

The PEG is discrete and does not interfere with speech or swallowing. Since the insertion of the PEG is a minor surgical procedure there is a common belief that it is harmless and has a low impact on daily life. Having the ability to stay at home may decrease clinical costs and even improve quality of care with supporting nutrition teams [8]. However, its benefits in clinical practice are not yet established [9,10]. There are qualitative disadvantages of having a PEG since it moves treatment from inpatient settings to the home, with a need for care from district nurses and general practitioners, sometimes with limited experience in dealing with problems regarding PEG and PEG feeding [8,11]. Moreover, it transfers treatment responsibility and activity to patients and their carers to a large extent. With enteral nutrition support, the social role of a meal disappears, removing all pleasure from mealtimes. This interference with social life seems to be of greater importance than PEG related problems of discomfort, leakage or blockage [12]. There is limited research exploring patients’ experiences of living with a PEG and to what extent support is needed. The aim of this study, therefore, was to increase the understanding of how to support patients that are condemned to live with a PEG by investigating patients’ experiences of living with a PEG.

## Methods

### Study design and data collection

A prospective cohort study was carried out at the Karolinska University Hospital in Stockholm, Sweden, during the period from 3 June, 2005 to 31 December, 2007. The data collection has been described in detail in a previous study [13]. In brief, all consecutive patients referred for PEG and had had their PEG for at least 2 months were eligible for inclusion in the study. Patients were excluded if they by any reason did not give their informed consent, did not understand the Swedish language, were too ill or could not communicate for other reasons. Baseline data at the time of PEG was collected prospectively through a predefined study protocol including information about patient characteristics (e.g., age, sex, marital status, education level) and clinical details (e.g., diagnosis, weight and height). At the 2-month follow-up, all patients remaining with a PEG were asked to complete a study specific questionnaire about their experience of living with a PEG. The questionnaire was tested for face validity in a number of patients and some minor changes were made. It included 16 questions of which we excluded six questions that were not consistent with our hypothesis. For the purpose of the current study we selected 10 questions assessing interference with daily activity, sleep, time-consumption, ability to influence the number of feeding occasions, feeling of confidence with self feeding, daily feeding, interference with oral intake and satisfaction. The response alternatives to all questions were 1) “not at all”, 2) “a little”, 3) “quite a bit” and 4) “very much”.

### The PEG procedure

A detailed description of the PEG procedure has been published elsewhere [13]. In brief, all patients were given oral as well as written information before the insertion of the PEG. The information included a description of the procedure, the brand name of the PEG catheter, detailed instructions of how to solve common problems associated with PEG, how to perform daily care of the wound site and the catheter, general nutrition advice and telephone numbers of the PEG outpatient clinic and of the dietician for contact whenever advice about the PEG device or nutrition would be needed. The PEGs were inserted by experienced surgeons assisted by experienced endoscopists.

### The PEG outpatient clinic

The care pathway for the PEG patients at Karolinska University Hospital is multidisciplinary and led by specially trained nurses, closely supported by experienced dieticians and physicians. All patients treated with PEG are followed up by these specially trained nurses at the PEG outpatient clinic 2 weeks, 2 months and 6 months after the PEG insertion. The patient can contact the nurses by telephone or book additional appointments whenever needed.

### Statistical analysis

For the purpose of this study all responses were dichotomized into “no” (response of “not at all” or “a little”) versus “yes” (response of “quite a bit” or “very much”). Two of the questions had descriptive response alternatives and were presented separately. For comparisons of patient characteristics and clinical variables, Fisher’s exact test was used to test for statistically significant differences at the 5 per cent level. Patients were stratified by sex, age (in two groups; <65 years and ≥65 years), marital status (in two groups; married or cohabitant and single), level of education (in two groups; public/high school and university) and by diagnosis (in two groups; cancer and neurological disease).

### Ethics

The patients and caregivers, or attending relatives, received oral and written information about the data collection and its use for research purposes, and the study was approved by the Regional Ethics Committee in Stockholm, Sweden.

## Results

### Patients

A total of 270 patients received a PEG during the study period. Within the 2-month follow-up period 51 patients died, 12 patients had the PEG removed, 3 patients were lost to follow-up and 55 patients were excluded according to the exclusion criteria without detailed information, leaving 149 patients eligible for the study. Among these, 147 patients (99%) were followed up with an appointment at the outpatient clinic 2 months after PEG and 104 (70%) responded to the questionnaire. Some characteristics of the study participants are presented in Table 1. The majority were men (64%) and the mean age was 64 years. Most patients were married (60%) and had no university education (60%). The indication for PEG was mainly due to a tumour (75%) or neurological disorders (23%), while only two patients were labeled “other causes” due to inflammatory diseases (myosit). More than a quarter of patients (28%) were underweight with a body mass index (BMI) below 20. The majority of patients used their PEG daily (81%) (Table 1). While about one third of the patients was not allowed to eat by mouth, the other patients could eat at least partly (Table 2).

**Table 1:** Characteristics of the 104 patients who had a percutaneous endoscopic gastrostomy (PEG) inserted and responded to a study specific questionnaire 2 months after PEG

	**Number**	**%**
**Gender**		
Male	67	64
Female	37	36
Age*		
< 65 years	54	52
≥ 65 years	50	48
**Marital status**		
Married	62	60
Single	31	30
Missing	11	11
**Education**		
Public school/High school	62	60
University	31	30
Missing	11	11
**Diagnosis**		
Cancer	78	75
Neurologic	23	22
Other	2	2
**BMI**		
< 20	29	28
≥ 20	71	68
Missing	4	4
**Enteral Nutrition via PEG**		
Daily	84	81
More seldom	10	10
Missing	10	10

* Cut off is based on the median age 64 years.

**Table 2:** Experience of living with a percutaneous endoscopic gastrostomy (PEG) among 104 patients who responded to a study-specific questionnaire 2 months after insertion

	**Number**	**%**
**Daily activity is limited due to the PEG**		
Not at all	50	48
A little	34	33
Quite a bit	14	13
Very much	5	5
Missing	1	1
**Disturbed sleep**		
Not at all	61	59
A little	38	37
Quite a bit	3	3
Very much	1	1
Missing	1	1
**PEG feeding is time consuming**		
Not at all	23	22
A little	39	38
Quite a bit	29	28
Very much	7	7
Missing	6	6
**Possibility to influence number of feeding times per day**		
Not at all	11	11
A little	18	17
Quite a bit	25	24
Very much	48	46
Missing	2	2
**Confidence with self feeding**		
Not at all	3	3
A little	3	3
Quite a bit	22	21
Very much	39	38
Missing	37	36
**Interfere with your oral intake**		
Not at all	38	37
A little	15	14
Quite a bit	4	4
Very much	4	4
Nil per os	31	30
Missing	12	12
**Satisfied with your PEG**		
Not at all	9	9
A little	11	10
Quite a bit	40	58
Very much	36	35
Missing	8	8

### Differences in the experience of PEG

Patients’ perspectives of living with a PEG for at least two months are presented in Table 2. In Table 3 the responses have been dichotomized as described in the methods above. Women reported a more negative experience of living with a PEG compared to men, however only the feeling of limitation in daily activity reached the level of statistical significance (p = 0.004). Older patients reported similar experiences as younger patients except that older patients (38%) experienced a more limited ability to influence the number of feeding times compared to younger patients (19%) (p = 0.026). No statistically significant differences were found between married and single patients. More highly educated patients found feeding to be more time-consuming (55%) than those with a public/high school education (23%) (p = 0.004). Patients with a cancer diagnosis found that the PEG feeding interfered with their oral feeding statistically significantly more than patients with a neurological disease (p = 0.009). Nearly 20% of all patients reported that they were not satisfied with having a PEG. There were six patients that had never used their PEG for nutrition support (data not shown).

**Table 3:** Experience of living with a percutaneous endoscopic gastrostomy (PEG) among 104 patients who responded to a study specific questionnaire 2 months after insertion in relation to personal characteristics

		**Gender**		**p**^**#**^	**Age**		**p**^**#**^	**Marital status**		**p**^**#**^	**Education**		**p**^**#**^	Diagnosis*		**p**^**#**^
	**Number (%)**	**Number (%)**			**Number (%)**			**Number (%)**			**Number (%)**			**Number (%)**		
	**Total**	**Male**	**Female**		**< 65**	**≥ 65**		**Married**	**Single**		**Public/high school**	**University**		**Cancer**	**Neuro-logical**	
																
	104 (100)	67 (64)	37 (36)		54 (52)	50 (48)		62 (60)	31 (89)		62 (60)	31 (30)		79 (76)	23 (22)	
**Daily activity is limited due to the PEG**				**#**												
No	84 (82)	60 (90)	24 (65)		43 (80)	41 (82)		52 (84)	23 (74)		52 (84)	23 (74)		64 (81)	19 (83)	
Yes	19 (19)	7 (10)	13 (35)		11 (20)	8 (16)		9 (15)	8 (26)		9 (15)	8 (26)		14 (18)	4 (17)	
**Disturbed sleep**																
No	99 (95)	65 (97)	34 (92)		51 (94)	48 (96)		59 (95)	30 (97)		58 (94)	31 (100)		76 (96)	21 (91)	
Yes	4 (4)	2 (3)	3 (8)		3 (6)	1 (2)		3 (5)	1 (3)		4 (6)	0 (0)		3 (4)	1 ( 4)	
**PEG feeding is time consuming**													#			
No	62 (60)	44 (66)	18 (49)		35 (65)	27 (54)		39 (63)	17 (55)		43 (69)	13 (42)		46 (58)	15 (65)	
Yes	36 (35)	20 (30)	16 (43)		15 (28)	23 (46)		19 (31)	12 (39)		14 (23)	17 (55)		27 (34)	8 (35)	
**Possibility to influence number of feeding times per day**							#									
No	29 (28)	15 (22)	14 (38)		10 (19)	19 (38)		16 (26)	10 (32)		20 (32)	6 (19)		19 (24)	9 (39)	
Yes	73 (70)	51 (76)	22 (59)		43 (80)	30 (60)		44 (71)	21 (68)		40 (52)	25 (81)		58 (73)	14 (61)	
**Confidence with self feeding**																
No	6 (6)	3 (4)	3 (8)		2 (4)	4 (8)		2 (3)	2 (6)		4 (6)	0		4 (5)	2 (9)	
Yes	61 (59)	43 (64)	18 (49)		39 (72)	22 (44)		37 (60)	21 (68)		35 (56)	23 (74)		55 (70)	5 (22)	
**Daily feeding**																
Yes	84 (81)	51 (76)	33 (89)		40 (74)	44 (88)		49 (79)	24 (77)		47 (76)	26 (84)		61 (77)	21 (91)	
No, more seldom	10 (10)	8 (12)	2 (5)		5 (9)	5 (10)		7 (11)	3 (10)		7 (11)	3 (10)		9 (12)	1 (4)	
**Interfere with your oral intake**																#
No	53 (51)	32 (48)	21 (57)		27 (50)	26 (52)		31 (50)	15 (48)		33 (53)	13 (42)		34 (43)	18 (78)	
Yes	39 (38)	26 (39)	13 (35)		19 (35)	20 (40)		22 (35)	13 (42)		20 (32)	15 (48)		34 (43)	4 (17)	
**Satisfied with your PEG**																
No	20 (19)	12 (18)	8 (22)		12 (22)	8 (16)		9 (13)	9 (29)		11 (18)	6 (19)		17 (22)	3 (13)	
Yes	76 (73)	51 (76)	25 (68)		38 (70)	38 (76)		49 (79)	20 (65)		46 (74)	23 (74)		55 (70)	19 (83)	

No = a response of not at all or a little, Yes = a response of quite a bit or very much. Due to missing answers, the number/frequencies of patients does not always add up to 104/100%.

* There are two persons with other diagnoses that are not presented here, # Statistical significance tested with Fisher’s exact test. p < 0.05.

### Contact support and feeding assistance

Table 4 presents patients’ choice of contact whenever a problem regarding the PEG occurred. The majority of patients (n = 83 including possible multiple responses) would turn to the PEG outpatient clinic with questions or problems regarding the PEG, followed by contact with the home care team (n = 15) and a dietician (n = 13). In Table 5, the results of patients’ responses to the question about feeding assistance are presented. The majority of patients responded that they fed themselves (n = 63 including possible multiple responses). The most common assistance was given by a spouse (n = 18) or by personnel at the nursing home (n = 10), and more seldom by district nurses (n = 5).

**Table 4:** Contacts regarding questions or problems concerning the percutaneous endoscopic gastrostomy (PEG) among 104 patients who responded to a study specific questionnaire 2 months after insertion

**Who do you contact with questions or problems concerning your PEG?**	**Total (n=104)**	**Male (n=67)**	**Female (n=37)**
	Number*		
PEG outpatient clinic	83	56	27
Home care team	15	8	7
Dietician	13	8	5
Primary care	9	6	3
Care staff at my nursing home	3	2	1
Other	2	1	1
Do not know	1	1	0

*Patients can choose more than one answer.

**Table 5:** Percutaneous endoscopic gastrostomy (PEG) feeding assistance among 104 patients who responded to a study specific questionnaire 2 months after insertion

**Who help you with your PEG feeding?**	**Total (n=104)**	**Male (n=67)**	**Female (n=37)**
	Number*		
Self care	63	44	19
Spouse	18	11	7
Care staff at my nursing home	10	6	4
District nurse	5	3	2
Home care team	4	3	1
Health care professionals at the hospital	3	2	1
Other relative	2	2	0

*Patients can choose more than one answer.

## Discussion

This exploratory study found that gender, age, education level and diagnosis are factors that might influence patients' experiences of living with a PEG, while marital status did not. This study also showed that patients preferred to contact the PEG outpatient clinic with problems about the PEG. Moreover, a majority of patients fed themselves with the PEG, but patients in need of assistance were mainly supported by their spouse and more seldom by district nurses.

Some methodological issues deserve attention. Our study period is limited to two months after the insertion of the PEG. A pre- and post period study should be interesting to paint a deeper light of the PEG experience. A threat to questionnaire surveys is selection bias due to non-participation or missing data. Although the response rate in the current study is relatively high (70%) there is a risk of selection bias. Therefore, the results should be interpreted with caution. The data was collected during ongoing clinical care, and the time with each patient was limited. It is sometimes difficult to get elderly people and patients with neurological diseases to respond to questionnaires due to cognitive impairments [14]. However, patients not capable of self-responding to the questionnaire and those not familiar with the Swedish language were excluded from participation in this study. These might be patients who are more vulnerable and care-dependent, at least in this early post-treatment period [15]. There were only 23% of patients with neurological disorders included in this study material indicating that a majority of the neurological patients are excluded because of their state and impairment. The use of a structured self-report questionnaire restricts a deeper knowledge about patients experience, however, it is easy to respond to and not as time-consuming as the use of an interview approach.

Previous literature investigating the experience of living with a PEG is limited. The tradition in the older generations where the woman takes responsibility for the preparation of food [16] might explain the feeling of limited daily activity among women in the current study. Sociologically, different roles are taken by gender, with different ways of perceiving symptoms and the illness process, with a sometimes over-estimation of morbidity in women. In fact gender differences with consistently worse results among women have often been described in previous studies[17].Our study confirms that the experience of living with a PEG was not affected by age except that the older patients experienced a decreased ability to influence the number of feeding times per day. Older patients may be more dependent on others than younger patients are [18] and this in turn might be a negative consequence of living with a PEG. Elderly patients with dependency on others seem to find it of great importance to be offered opportunities for living life as usual [19]. Patient satisfaction with care depends on the health problem for which the patient is being treated, but is generally high. Personal matters such as education level may however affect patients’ perceptions of satisfaction with care. It has been concluded that satisfaction with care is higher in patients with a low level of education [20] which is in line with the results of the present study where patients with a university education found feeding to be more time-consuming than those with public or high school education. The fact that every fifth patient was dissatisfied with the PEG indicates a need for careful information about living with the PEG, before insertion of the PEG.

In order to maintain nutritional intake, PEG is often required for cancer patients during a restricted oncological treatment early in the care pathway, sometimes even before the need for enteral nutrition support, and for a limited period [21-23], while neurological patients most likely require a PEG later in the care pathway and will live with it for the rest of their lives. Once they agree to receive a PEG, they are perhaps more sympathetic to it. A PEG is suggested to be a milestone in the palliative care of ALS patients [24,25] but the fact that 30% of the patients were underweight (BMI below 20) at the time of PEG in the current study might indicate the PEG insertion to be rather late in the care pathway for some patients. Clinical benefits for head and neck cancer patients are inconclusive [10]. Terrell et al. [26] found that the presence of a gastrostomy among head and neck cancer patients was associated with the statistically lowest scores in quality of life based on the SF-36 questionnaire, and suggest that gastrostomy is a constant reminder of the cancer disease during treatment. Cancer patients in the current study found the PEG feeding interfered with their oral intake to a larger extent than neurological patients did, but unfortunately there is not much written about these potential different experiences of living with a PEG when comparing diagnoses [27], but this is probably of great importance for health care professionals to keep in mind. Whatever the patients experience is of living with a PEG, enteral nutrition sometimes is the only alternative for sufficient nutrition.

The major PEG-related problems might not be discomfort, leakage or blockage, but rather interference with family life and social activities [12]. This might at least partly explain the experience of time-related problems described in this study. Previous research has reported that home care responsibility today is transferred to a larger extent to the patient and their relatives instead of primary care [11,19]. This is supported by the results from the current study that when needed, the patient was most often assisted by a spouse and more seldom by a district nurse. Since caring for PEG patients is time-consuming (up to 15 hours per week) [28] it may place a burden on family members by transferring the responsibility from primary care to the patient and their family [29].

It is important that the health care providers facilitate the insertion of PEG, increase the effectiveness of patient counseling and monitoring for complications and to inform and discuss the possibility to remove of the PEG [30]. The finding that a large group of patients preferred to turn to the PEG outpatient clinic with questions or problems regarding the PEG and feeding, highlights this need for an outpatient clinic with specialized knowledge in PEG care, including a multidisciplinary team for referral of specific problems [31].

Previous research has shown that regular systematic nutrition team follow-up for gastrostomy-fed patients does not increase costs and may improve quality of care [8]. Therefore it can be recommended that all patients receiving a PEG should have access to an outpatient clinic with specialized knowledge in PEG care. There are several ways to improve the life of patients living with a PEG. Kurien et al. shows in a large prospective study that dietetic aftercare community service reduce hospital readmissions [32]. This is not surprisingly since Brotherton et al. found in semi-structured interviews with PEG patients that issues that was emergent related to the enteral feeding were disturbed sleep, limited ability to go out, limited choice of clothing, difficulties finding feeding places. [29] By contact with a dietician during the care pathway, support with enteral feeding can be arranged. Enteral formulas must not only suit the patient’s specific nutritional needs, but might also suit the number of feedings per day and speed time for feeding. Moreover, the dietician can together with the patient and their relatives arrange the best suited device for the PEG feeding, as there is a range of devices for administration of enteral nutrition. For instance, mobile pumps and carrying packs with related giving sets can offer a more mobile life than drip stands do. At the time of discharge from hospital it is important that the health care professional at the hospital ward reports the patient to a district nurse. Health care professionals working in primary care are encouraged to collaborate and get support from the PEG outpatient clinic for the further care of the patient at home. This would make the district nurse feel more secure even if inexperienced in caring for problems regarding PEG and PEG feeding [8,11,33].

## Conclusion

In conclusion, PEG is sometimes the only alternative to enable sufficient nutritional support but is time-consuming and interferes with the daily life of the patient. Although a vast majority of patients are satisfied with their PEG, the experiences may be dependent on personal characteristics such as age, sex, education and the diagnosis. Family members are the main carers for PEG patients at home and if problems with the PEG arise, most patients turn to the PEG outpatient clinic for help.

## Competing interests

The authors declare that they have no competing interests.

## Authors' contributions

LM, JB and PL formulated the study hypothesis. LM and JB recruited the patients. LM and PL conducted the statistical analyses and LM, JB and PL interpreted the results. LM drafted the manuscript and JB and PL provided inputs. All authors read and approved the final version of the manuscript. PL provided funding.

## Financial support

This study was supported by the Swedish Cancer Society and through the regional agreement on medical training and clinical research (ALF) between Stockholm County Council and Karolinska Institutet.

## Pre-publication history

The pre-publication history for this paper can be accessed here:

http://www.biomedcentral.com/1471-230X/12/126/prepub
